# In Search of Spectroscopic Signatures of Periodontitis: A SERS-Based Magnetomicrofluidic Sensor for Detection of *Porphyromonas gingivalis* and *Aggregatibacter actinomycetemcomitans*

**DOI:** 10.1021/acssensors.1c00166

**Published:** 2021-04-01

**Authors:** Evelin Witkowska, Anna M. Łasica, Krzysztof Niciński, Jan Potempa, Agnieszka Kamińska

**Affiliations:** † Institute of Physical Chemistry, Polish Academy of Sciences, Kasprzaka 44/52, 01-224 Warsaw, Poland; ‡ Department of Bacterial Genetics, Institute of Microbiology, Faculty of Biology, University of Warsaw, Miecznikowa 1, 02-096 Warsaw, Poland; § Department of Microbiology, Faculty of Biochemistry, Biophysics, and Biotechnology, Jagiellonian University, Gronostajowa 7, 30-387 Krakow, Poland; ∥ Oral Immunology and Infectious Diseases, University of Louisville School of Dentistry, 501 S. Preston Street, Louisville, Kentucky 40202, United States

**Keywords:** surface-enhanced Raman spectroscopy (SERS), principal component analysis (PCA), Porphyromonas gingivalis, Aggregatibacter actinomycetemcomitans, silver-coated magnetic nanoparticles (Fe_2_O_3_@AgNPs), periodontitis, Alzheimer’s disease

## Abstract

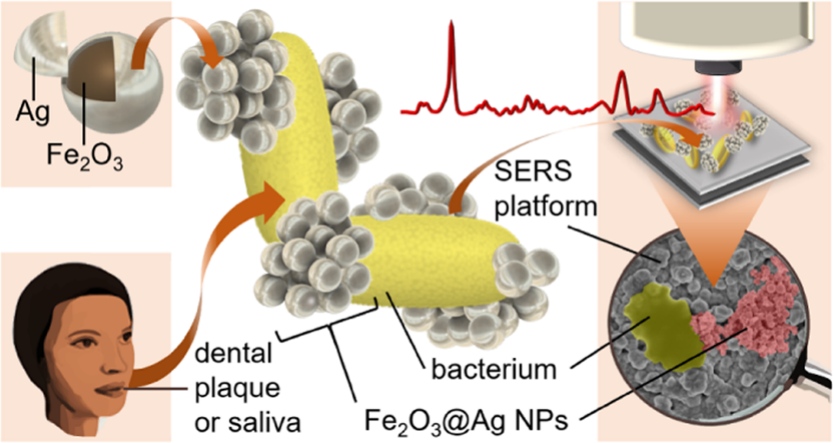

Recently, *Porphyromonas gingivalis*, the keystone pathogen implicated in the development of gum disease (periodontitis), was detected in the brains of Alzheimer’s disease patients, opening up a fascinating possibility that it is also involved in the pathobiology of this neurodegenerative illness. To verify this hypothesis, an unbiased, specific, and sensitive method to detect this pathogen in biological specimens is needed. To this end, our interdisciplinary studies demonstrate that *P. gingivalis* can be easily identified by surface-enhanced Raman scattering (SERS). Moreover, based on SERS measurements, *P. gingivalis* can be distinguished from another common periodontal pathogen, *Aggregatibacter actinomycetemcomitans*, and also from ubiquitous oral *Streptococcus* spp. The results were confirmed by principal component analysis (PCA). Furthermore, we have shown that different *P. gingivalis* and *A. actinomycetemcomitans* strains can easily adsorb to silver-coated magnetic nanoparticles (Fe_2_O_3_@AgNPs). Thus, it is possible to magnetically separate investigated bacteria from other components of a specimen using the microfluidic chip. To obtain additional enhancement of the Raman signal, the NPs adsorbed to bacterial cells were magnetically attracted to the Si/Ag SERS platform. Afterward, the SERS spectra could be recorded. Such a time-saving procedure can be very helpful in rapid medical diagnostics and thus in starting the appropriate pharmacological therapy to prevent the development of periodontitis and associated comorbidities, e.g., Alzheimerʼs disease.

Surface-enhanced Raman spectroscopy (SERS) is a technique allowing us to obtain strong amplification of the Raman scattering effect on analyzed molecules, which are localized in “hot spots”, the crevices of the metal nanostructures characterized by very strong local field enhancement caused by the surface plasmon resonance.^1,2^ The mentioned nanostructures are usually made of SERS-active metals, such as silver, gold, copper, or their alloys.^3^ The reason for using these metals specifically is associated with the fact that they have localized surface plasmon resonance (LSPR) that covers most of the visible and near-infrared wavelength ranges. This, in turn, means that they are suitable for use with visible and NIR laser systems commonly applied in Raman measurements.^4^

The SERS technique is also known for its high level of sensitivity and specificity, which allows for the detection, identification, and characterization of various chemical compounds or biological materials. As a result, one may obtain a specific and unique fingerprint of the compound/material under study. The SERS effect relies on the combination of two mechanisms, electromagnetic (EM)^5,6^ and chemical (charge-transfer, CT),^7,8^ that can together lead to the amplification of the Raman signal even by 12 orders of magnitude in comparison to normal Raman spectroscopy.^9^ Such enhancement of inherently weak Raman bands allows us to characterize single molecules^10^ or detect and identify the samples of biological origin.^11^

Huge sensitivity, high selectivity, and the possibility to perform rapid, label-free, and nondestructive analysis lead to a wide range of practical applications of the SERS technique. Currently, the technique is used in biomedical, analytical, and environmental studies, as well as in forensic science and the food industry. Recent progress in the field of preparation of SERS substrates^12,13^ has led to a large array of ready-to-use and commercially available products for a variety of applications. New approaches allowed also for the cost reduction of SERS platform preparation. Additionally, high-tech Raman spectrometers equipped with various laser lines have become more accessible due to substantial miniaturization and lower purchase costs while maintaining high measurement parameters.

As a result, the interest in SERS detection and identification of biological samples such as proteins, viruses, fungal cells, and cancer cells has increased significantly.^14−19^ Moreover, numerous scientific publications have proven that SERS can be applied for reliable identification and detection of various bacterial strains.^20−25^ This is an important issue, especially when considering bacterial pathogens responsible for different human diseases.

In this work, we focused on *Porphyromonas gingivalis*, a Gram-negative, anaerobic bacterium found in the human oral cavity and responsible for chronic periodontitis.^26,27^ We compared the obtained results for *P. gingivalis* with the spectra of *Aggregatibacter actinomycetemcomitans* and two streptococcal species. Of note, *A. actinomycetemcomitans* is recognized as the causative agent of a rare form of gum disease, aggressive periodontitis. Chronic periodontitis is a complex disorder resulting from dynamic interactions between pathogens, other oral microbiota, and the host immune system and is the second most prevalent oral disease, right after dental caries. Genetic, environmental, and behavioral factors such as immunodeficiencies, diabetes, poor oral hygiene, and cigarette smoking, to name a few, are also important for the course of the disease. Infection-driven inflammation leads to swollen and bleeding gums, degradation of periodontal ligament, resorption of alveolar bone, periodontal pocket formation, and eventually to teeth loss.^28,29^

Chronic periodontitis is a slowly developing pathology, not easy to diagnose, and is challenging to treat. Although it brings discomfort, pain, chewing difficulties, and eventually teeth exfoliation (if not treated), it is also associated with some serious systemic diseases such as rheumatoid arthritis, atherosclerosis, aspiration pneumonia, or preterm birth.^30,31^ Moreover, the periodontal pathogens are implicated in the development of cancer,^32^ and, recently, *P. gingivalis* was directly implicated as a putative contributor to the pathogenesis of Alzheimer’s disease. It was shown that *P. gingivalis* virulence factors, gingipains, were present in patients’ brains.^33^ Additionally, the bacterial colonization of the brain and the cerebrospinal fluid was detected through the polymerase chain reaction (PCR) technique.^33^^33^

Herein, we report an independent culturing method allowing for fast, specific, and sensitive detection of *P. gingivalis*, *A. actinomycetemcomitans*, and *Streptococcus* spp. We demonstrated that it is possible to obtain the intense and unique SERS spectra of investigated bacterial cells. Moreover, the principal component analysis (PCA) allowed us to separate the SERS spectra of bacteria belonging to different species (from 82 to 91% accuracy, depending on the type of the analyzed sample). Importantly, unique SERS spectra were clearly observed for different strains of *P. gingivalis* and *A. actinomycetemcomitans*, thus making it possible to distinguish one strain from another.

We also proved that the SERS method coupled with the microfluidic technique and magnetic separation allows us to detect and identify *P. gingivalis* and *A. actinomycetemcomitans* strains in liquid samples such as human saliva. For this purpose, the bacterial cells were mixed with Fe_2_O_3_@Ag magnetic nanoparticles (NPs) in a microfluidic chip. The created bacteria–NP aggregates were next attracted by a neodymium magnet placed under the Si/Ag platform. Owing to the use of both silver-coated NPs and a Si/Ag substrate, the greatly enhanced SERS signal, characteristic for each of the investigated bacterial species/strains, was obtained. The conducted experiments and results are the first step on the path to developing the method, which could be applied for reliable assessment of microbiological risks underlying the development of periodontitis and associated systemic comorbidities, such as rheumatoid arthritis and Alzheimer’s disease.

## Experimental Section

### Bacterial Strains

Five strains of *P. gingivalis* [33277 (wt), W83 (wt), ΔK/ΔRAB-A, ΔPorN, AL022] were obtained from the Institute of Microbiology, Faculty of Biology, University of Warsaw, Poland. The construction of *P. gingivalis*-mutated strains (ΔK/ΔRAB-A, ΔPorN) was reported in the publication of Maria Rapala-Kozik et al.,^34^ whereas generation of AL022 is described in the Supporting Information (Figure S1). Three strains of *A. actinomycetemcomitans* [HK1651 (wt), JP2, 652 (wt)] were a kind gift from Prof. Donald R. Demuth (University of Louisville School of Dentistry, Louisville, Kentucky, United States), while two streptococcal strains, namely*, Streptococcus pseudopneumoniae* 6178/12 and *Streptococcus mitis* 3705/04, were donated by Prof. Anna Skoczyńska (the National Reference Centre for Bacterial Meningitis in the National Medicines Institute, Warsaw, Poland). Relevant genotype/phenotype or origin information of all used strains is given in Table 1.

**Table 1:** Genotype and Phenotype Information of All Strains Used in the Study

strain	relevant genotype/phenotype/origin	source
*P. gingivalis*		
ATCC 33277	wild type	(35)
W83 (ATCC BAA-308)	wild type; altered in fimbriae production	(36)
ΔK/ΔRAB-A	W83*: kgp, rgpA, rgpB* (Tet^r^Cm^r^Em^r^); devoid of all gingipains	(34)
ΔPorN	W83: *porN* (Em^r^); defective in T9SS (type IX secretion system)	(37)
AL022	ATCC 33277: Δ*PGN_1642* (Em^r^), gene of unknown function	present study
*A. actinomycetemcomitans*		
HK1651	wild type; serotype b, highly leukotoxic, rough colony	(38)
JP2	wild type; serotype b, smooth colony, highly leukotoxic derivative of HK1651	(38, 39)
652	wild type; serotype c, minimally leukotoxic	(39)
*Streptococcus* spp.		
*S. pseudopneumoniae* 6178/12	human bronchial secretion	(40)
*S. mitis* 3705/04	human sputum	(40)

#### *P. gingivalis*

In the present study, two sets of *P. gingivalis* strains were used. (1) the W83 wild-type strain and its two derivatives, mutants with altered virulence, namely, *ΔK/*Δ*RAB-A* (Δ*kgp/rgpA/rgpB*) and Δ*porN*. (2) The ATCC 33277 wild-type strain and its mutated progeny AL022 (Δ*PGN_1642*) of an unknown phenotype.

The main difference between W83 and ATCC 33277 wild-type strains is that W83 is coated with a polysaccharide capsule and devoid of functional fimbriae.^41^ Similar features may be expected for *ΔK/*Δ*RAB-A* and Δ*porN* as they both show changes in the cell surface structure displayed by the greatly reduced and entirely absent electron-dense surface layer, respectively.^42,43^ The first phenotype is due to the lack of gingipains (Kgp, RgpA, and RgpB), the main virulence factors usually attached to the cell surface. The second phenotype is caused by the deficiency in T9SS, a secretion system transporting various proteins, including gingipains, across the membranes and subsequently attaching them to the cell surface.^44^

#### *A. actinomycetemcomitans*

The three *A. actinomycetemcomitans* strains analyzed in the present work are representatives of two serotypes: b, highly leukotoxic (strains HK1651 and JP2) and c, minimally leukotoxic (strain 652). Additionally, within serotype b, the strains HK1651 and JP2 show surface structure differences described as rough and smooth colonies, respectively. This is due to a spontaneous mutational event within the fimbria biogenesis operon.^38,39^

#### Streptococci

In the present study, two different streptococcal species were used: *S. mitis* 3705/04, which inhabits the human mouth, and *S. pseudopneumoniae* 6178/12, which is the causative agent of pneumonia in humans and can also be found in the human oral cavity.

### Culture Media and Growth Conditions of Bacterial Strains

*P. gingivalis* strains were grown in enriched BD BBL trypticase soy broth (eTSB per liter: 30 g of trypticase soy broth, 5 g of yeast extract, 5 mg of hemin, pH 7.5; 0.5 g of l-cysteine and 2 mg of menadione) and on eTSB blood agar (eTSB medium containing 1.5% agar and 5% defibrinated sheep blood) at 37 °C in anaerobic conditions using an Anoxomat Mark II system (90% nitrogen, 5% carbon dioxide, and 5% hydrogen). Cultures of mutated strains were additionally supplemented with appropriate antibiotics (erythromycin at 5 μg/mL and/or tetracycline at 1 μg/mL and chloramphenicol at 3.5 μg/mL).

*A. actinomycetemcomitans* strains were grown in BD BBL brain heart infusion (BHI) broth and on BHI agar (1.5% agar), both supplemented with 40 mg/L NaHCO_3_, at 37 °C in microaerobic conditions using an Anoxomat Mark II system (85% N_2_, 10% CO_2_, 5% O_2_), while streptococcal strains were cultured on Columbia blood agar at 37 °C in aerobic conditions.

All bacteria were grown to the early stationary phase (ca. 24 h in broth or 48 h on plates in the case of *P. gingivalis* and *A. actinomycetemcomitans* or ca. 16 h on plates in the case of *S. pseudopneumoniae* and *S. mitis*).

The bacterial strains belonging to *P. gingivalis* and *A. actinomycetemcomitans* were cultured both on solid (agar) and in a liquid medium to check if the culture conditions are altered for the SERS measurements.

### Human Saliva

The human saliva used in the experiments was obtained from a healthy male volunteer. Before experiments, the saliva was placed in the syringe and filtered by applying syringe filters with pore sizes of 0.2 μm. The performance of experiments was in accordance with the institutional guidelines and relevant laws and was approved by the Ethics and Bioethics Committee of Cardinal Stefan Wyszyński University in Warsaw.

### Sample Preparation for SERS Measurements

**Figure 1:**
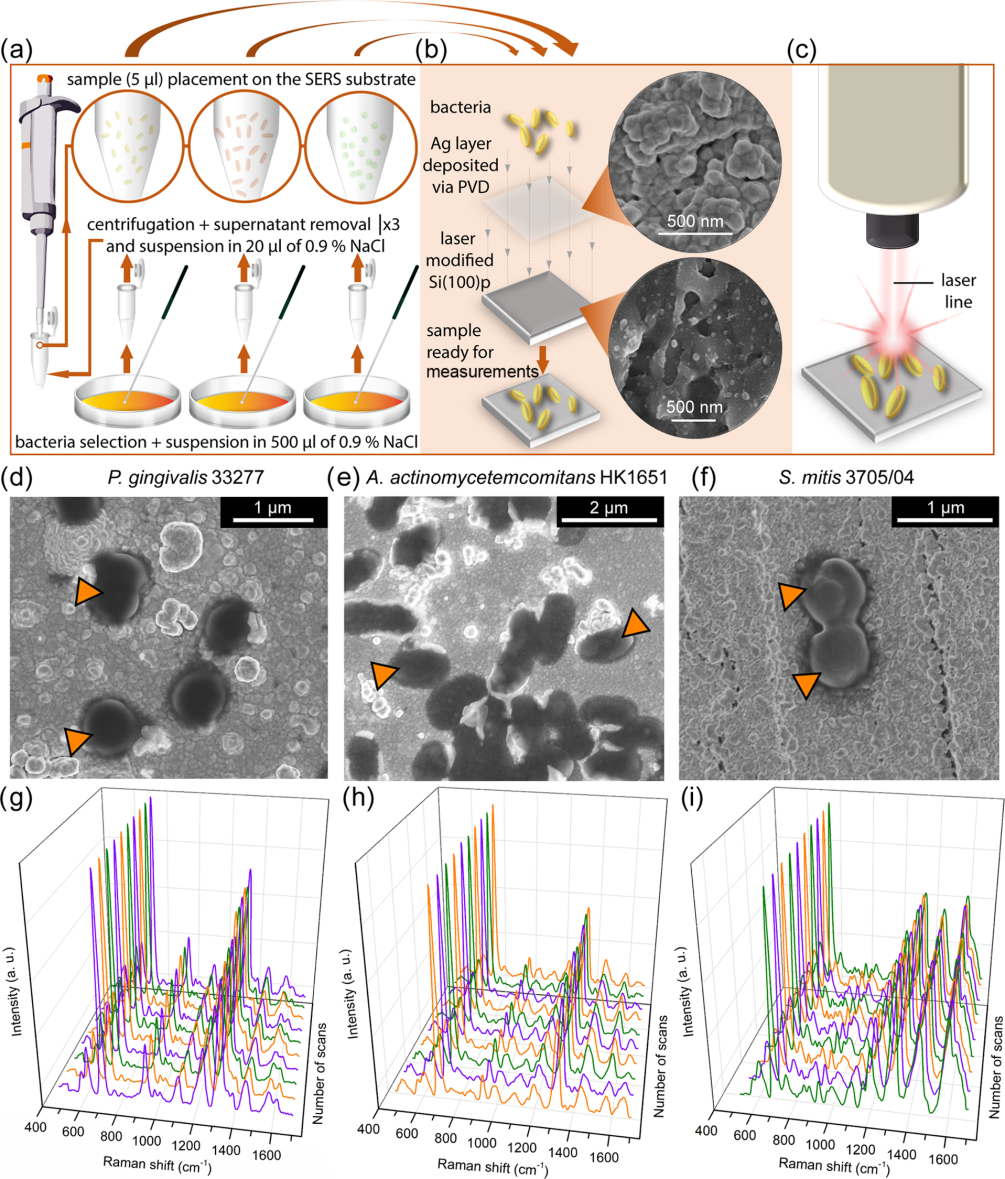
Preparation of the sample for SERS experiments, SEM imaging, and reproducibility of the SERS spectra. The experiment comprised preparation of bacterial cell samples (a), preparation of the Si/Ag SERS substrate (b), and SERS measurement of bacterial cells (c). (d–f) SEM images of three selected bacterial strains placed over the Si/Ag SERS platforms. (g–i) 3D plots showing the reproducibility of the SERS measurements performed for three selected bacterial strains. Orange arrowheads in all SEM images (d–f) indicate bacterial cells adsorbed to the Si/Ag platform.

In the case of bacteria cultured on solid (agar) media, about three single bacterial colonies were selected and suspended in 500 μL of a sterile saline solution (0.9% NaCl) via an inoculation loop. Then, bacteria were gently mixed with the saline solution by an automatic pipette. In the case of bacteria grown in liquid media, 500 μL of each culture was placed in a 1.5 mL tube. Next, both types of samples were centrifuged (5 min, 1070*g*), the supernatants were discarded, and the pellets were suspended in 500 μL of the sterile saline solution. The washing procedure was repeated three times. The concentration of bacteria in each sample was at the level of 10^8^ cfu/mL, as the cell suspension was equivalent to the 0.5 McFarland turbidity standard (McFarland densitometer DEN-1B, Biosan).^45^ After the final centrifugation step and supernatant removal, the pellets were suspended in 20 μL of the sterile saline solution. About 5 μL of each mixture was used for SERS measurements by placing it onto the SERS substrate 5 min before measurement (to let the sample dry). Each strain was cultured independently three times, both on solid (agar) and in liquid media. The scheme of the sample preparation for the SERS measurements is depicted in Figure 1a–c.

### Instrumentation: SERS Spectroscopy, Scanning Electron Microscopy (SEM), and X-ray Diffraction (XRD)

To perform SERS measurements, a Bruker’s BRAVO spectrometer equipped with a Duo LASER (700–1100 nm) and a CCD camera was used. The spectrometer also has a function of fluorescence rejection, which was applied during all measurements. The laser power for both lasers was <100 mW, while the spectral resolution was 2–4 cm^–1^. The SERS spectra were recorded repeatedly for 30 min. A single measurement, composed of three accumulations, each of 6 s, was completed after approximately 45 s (because of data processing and transfer). The results were next processed via OPUS software, ver.2012 (Bruker Optic GmbH, Germany). All of the spectra presented in the article were averaged from at least 25 single measurements.

After SERS experiments, the SERS substrates with bacteria placed on them were subjected to SEM measurements, without prior treatment of the cells. The substrates were attached to aluminum SEM pin stubs with conductive liquid silver paint (Pelco, Ted Pella, Inc.). The SEM measurements, performed under high vacuum, were obtained using an FEI Nova NanoSEM 450 scanning electron microscope. The range of the accelerating voltage was 2–10 kV.

To assess the sizes of silver nanostructures building Si/Ag substrates applied in the study, the X-ray diffraction (XRD) technique was used. The samples were analyzed using an Empyrean 2 (PANalytical) X-ray Diffraction System [radiation line Cu Kα_1_ (KL3) was 1.540598 Å and the analyzed reflex Ag(111) was at an angle of 2θ = 38.116°].

### Preparation of SERS Substrates

#### Silicon/Ag Platforms (Si/Ag Platforms)

The preparation of the silicon-based SERS substrate consists of five main steps. First, the silicon wafer was cut into squares (3.5 × 3.5 mm^2^) using a mechanical cutting saw (type: Disco DAD 2H/6TM) at the Institute of Electronic Materials Technology in Warsaw, Poland. Second, the silicon surfaces were washed with great care with deionized water. Next, all of the residues were removed and the cut wafers were dried in a stream of argon. Afterward, the silicon surface was modified with the use of a femtosecond laser (based on potassium yttrium tungstate, with 1030 nm wavelength, repetition rate 300 kHz, and a single pulse set to 300 fs). In the final step, the modified silicon surface was sputtered with 100 nm of silver (the target was purchased from Mennica Metale Szlachetne, Poland) via physical vapor deposition (PVD) (sputtering device: Quorum, Q150T ES, Laughton, U.K.). The vacuum during the process was at the level of 10^–2^ mbar, the sputtering current was 25 mA, and the process took place in an argon plasma atmosphere. The final thickness of the silver layer was controlled with a quartz crystal monitor. The SEM and XRD analyses of the Si/Ag substrates are presented in the Supporting Information (Figure S2a,b).

#### Silver-Coated Fe_2_O_3_ (Fe_2_O_3_@Ag) Magnetic Nanoparticles

The synthesis procedure of Fe_2_O_3_@Ag magnetic nanoparticles was inspired by a previously reported method^46^ and modified. The developed synthesis pathway is a one-step thermal decomposition of silver acetate salt in the presence of iron(III) oxide nanoparticles. Fe_2_O_3_ nanospheres having an average diameter of less than 50 nm (Merck KGaA, Darmstadt, Germany) were initially grated in an agate mortar with silver acetate (Merck KGaA, Darmstadt, Germany) in a mass ratio of 1:10. The mixture obtained in this way was transferred to plastic containers, and agate balls were added to it. Subsequently, the substrates were ground for 30 min in a Retsch MM400 mixer ball mill with a frequency of 20 Hz. The obtained powder was transferred to a round-bottom boat ceramic crucible and placed in a Carbolite tube furnace (Keison Products, Chelmsford, Essex, U.K.). The mixture was heated up to 200 °C at a heating rate of 10 °C/min, and then, it was kept in a nitrogen atmosphere at 200 °C for 2 h. The furnace was cooled to ambient temperature with the product kept in a protective nitrogen atmosphere. Next, the product was purified by suspending it in 500 mL of distilled water. Additionally, an ultrasonic bath was applied at this stage. Finally, the suspension was poured over the neodymium magnet till the supernatant became transparent. The final product was dried at ambient temperature because of which the nonmagnetic phase and all of the residuals from the synthesis were removed from the product in the supernatant phase. The SEM analysis and size distribution histogram of Fe_2_O_3_@AgNPs are presented in the Supporting Information (Figure S2c,d).

### Design and Implementation of the Microfluidic Chip

The layered diagram of the microfluidic system integrated with the magnetically controlled area containing the SERS-active platform is depicted in Figure S3a. The system included a quartz glass with dimensions of 20 × 20 mm^2^, which allowed us to collect signals during the experiment. The microfluidic system was designed with the use of MasterCAM software and milled in a 5- and 2 mm polycarbonate (PC) slab (Bayer) with a computer numerical-controlled (CNC) milling machine (ErgWind, type MFG4025P). To join two main milled elements, PC slabs were pressed together at a high temperature (*T* = 130 °C) for 30 min. The top cover was connected to the rest of the chip with screws and sealed with an O-ring. A technical drawing with dimensions is shown in Figure S3b.

An infusion pump system (Harvard Apparatus Pump Series, MA) was used for the automated control of the flow. For the injection of the analyte and the buffer into the microfluidic system and collection of the separated fractions, blunt-ended needles with an outer diameter of 0.8 mm were installed in the holes that were drilled in appropriate places in the plates. Polyethylene (PE) tubings with an inner diameter of 0.8 mm were applied to connect the chip with an infusion syringe pump and a residue container.

## Results and Discussion

### SEM Images and Reproducibility of SERS Measurements

In the present study, two pathogenic bacterial strains (*P. gingivalis* and *A. actinomycetemcomitans*) were included to show the spectroscopic differences between them. Additionally, the differentiation between *P. gingivalis* and nonpathogenic streptococci (*S. mitis*, *S. pseudopneumoniae*) is presented. Such a distinction is of utmost importance, as all mentioned bacterial species can be found on the tooth surface and in the saliva; however, only *P. gingivalis* infection has been linked to i.a. rheumatoid arthritis and Alzheimer’s disease.

The surface of the SERS substrate was visualized via SEM imaging, which allowed us to study the morphology of Si/Ag platforms and demonstrate the adsorption of investigated bacterial cells to the surface of these platforms (see Figure 1d–f). The silicon plates covered with silver grains allowed for the enhancement of the Raman signals of studied bacterial strains and to obtain reproducible SERS spectra (see Figure 1g–i). As can be noticed, the individual SERS spectra of the same strain obtained by repeated scans are very similar to each other. On the other hand, when comparing the SERS spectra of different species, clear dissimilarities concerning band locations and intensities are observed. This proves that SERS substrates used in this study are appropriate to conduct SERS measurements of microbiological samples.

### Investigation and Chemometric Analysis of Bacterial SERS Spectra Obtained on Si/Ag SERS Platforms

#### SERS Spectra of *P. gingivalis*

**Figure 2:**
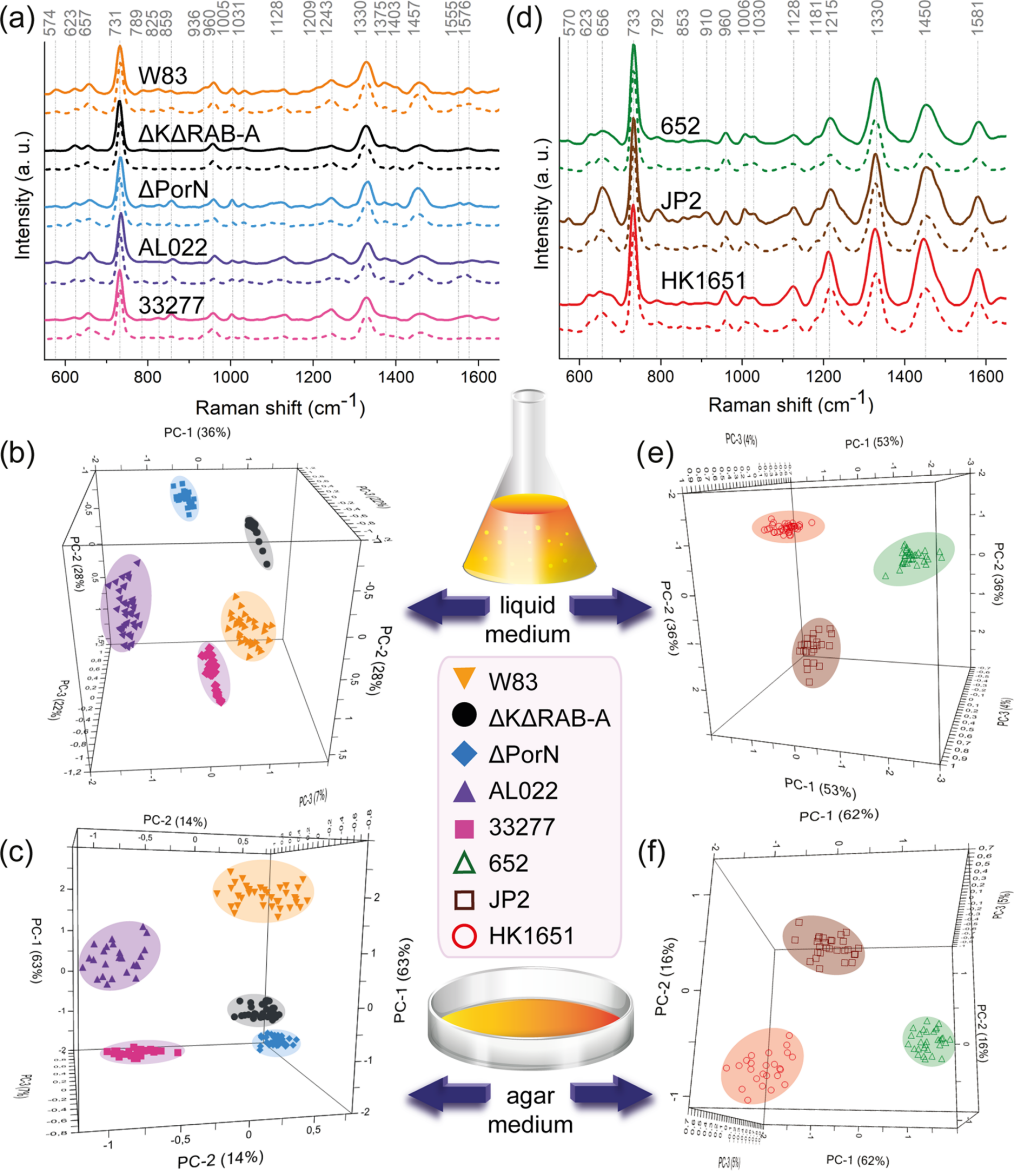
SERS spectra (a, d) and three-dimensional (3D) PCA plots (b, c, e, f) obtained for five investigated *P. gingivalis* strains (W83, ΔK/ΔRAB-A, ΔPorN, AL022, and 33277) and three *A. actinomycetemcomitans* strains (652, JP2, and HK1651). Spectra marked with solid lines in the images (a, d) and PCA results from the images (b, e) show results obtained for bacteria grown on agar media, while spectra marked with dashed lines in the images (a, d) and PCA results from the images (c, f) show results obtained for bacteria grown in liquid media.

To check the ability of the SERS technique to differentiate strains of the same bacterial species, five strains of *P. gingivalis* were tested. The average results of the obtained SERS spectra, in the range from 550 to 1650 cm^–1^, are depicted in Figure 2a (the spectrum averaged over all *P. gingivalis* strains is depicted in Figure S4a,b). As one can notice, the spectra of all investigated strains show a high degree of similarity; each spectroscopic image is dominated by the bands at ca. 730 and 1330 cm^–1^, which can be assigned to the in-plane ring breathing mode of adenine and to CH_3_/CH_2_ wagging, respectively.^47,48^ The remaining observed bands can be attributed to metabolites of purine degradation other than adenine and AMP, e.g., guanine, xanthine, hypoxanthine, and uric acid,^49^ and to bacterial cell wall components, e.g., phospholipids^50^ and proteins.^51^

The minute differences between spectra of studied strains may be attributed to the presence/absence of some antigens and/or secreted chemical substances, and to the metabolic profile of bacteria.^49−51^ This, in fact, applies when comparing any bacterial strains, irrespective of whether they are assigned to one or various species. The minor differences are also observed for the same bacterial strain, depending on whether it is cultured in the planktonic form (dashed lines in Figure 2a) or on agar plate (solid lines in Figure 2a). This may be associated with the culture conditions; as bacterial cells do not change locations on solid (agar) medium, they are constantly affected by metabolites secreted by them and other cells present in the same colony. This is not the case in liquid cultures incubated with shaking, as every bacterial cell constantly changes its location. As the SERS technique is known for its sensitivity at the level of a single molecule, the mentioned differences can be noticed in the SERS spectra of investigated bacteria. For this reason, it is very important to ensure identical measurement parameters and culture conditions for all compared bacterial strains. Nonetheless, despite the described dissimilarities, the main features characteristic for *P. gingivalis* species are still visible, especially the band at 1243 cm^–1^ assigned to amide III vibrations.

#### PCA Performed for SERS Spectra of *P. gingivalis*

The differences or similarities between the SERS spectra of *P. gingivalis* strains can be easily presented via principal component analysis (PCA). Figure 2b,c demonstrates 3D-PCA separating five *P. gingivalis* strains cultured on solid (agar) and in liquid media, respectively. As can be noticed, the first three principal components (PC-1, PC-2, and PC-3) are responsible together for 86% of the variance in the data while comparing bacteria grown on agar media (Figure 2b) and for 84% of the variance in the data in the case of liquid cultures (Figure 2c). Such a result indicates that the SERS method is useful even when comparing strains similar from phenotypical, physiological, and biochemical points of view.

#### SERS Spectra of *A. actinomycetemcomitans*

Furthermore, to determine whether the SERS technique provides the possibility of distinguishing *P. gingivalis* from other bacterial strains associated with localized aggressive periodontitis, additional SERS measurements were performed, this time on three strains of *A. actinomycetemcomitans*. As previously performed, the measurements were conducted on bacteria grown on agar plates (solid lines in Figure 2d) and in the planktonic form (dashed lines in Figure 2d). The results averaged over all investigated *A. actinomycetemcomitans* strains are presented in Figure S4c,d. As can be observed, all of the spectra are very similar to each other, regardless of the strain and growth conditions. Similar to *P. gingivalis*, the spectra are dominated by the bands at around 733 and 1330 cm^–1^. Additionally, one can see strong bands at ca. 1215, 1450, and 1581 cm^–1^, which can be assigned to C–N stretching/amide III/thymine, CH_3_/CH_2_ deformation, and the C=C bending mode of phenylalanine, respectively.^48^ There are also numerous bands of lower intensities, e.g., at 623 cm^–1^ (C–C twisting in protein),^51^ 656 cm^–1^ (C–S stretching, hypoxanthine, xanthine, guanine),^52,53^ 960 cm^–1^ (C=C deformation, adenine, guanine, NAD^+^),^54−56^ 1006 cm^–1^ (phenylalanine, C–C aromatic ring stretching),^54−56^ 1128 cm^–1^ (=C–O–C= in unsaturated fatty acids in lipids),^56^ and 1181 cm^–1^ (tyrosine).^53^ As these bands are reproducible in all individual SERS measurements, they apparently represent an inherent feature of tested bacterial strains and also contribute to group separation in PCA. Such defined band locations and band intensities, which are characteristic for specific bacterial species, are very important for their taxonomic affiliation determined by the SERS technique.

#### PCA Performed for SERS Spectra of *A. actinomycetemcomitans*

The PCA results obtained for the three strains of *A. actinomycetemcomitans* are presented in Figure 2e,f. The PC-1, PC-2, and PC-3 values together yielded 93% of the total variance in the case of strains cultured on agar plates and 83% when analyzing bacteria grown in the liquid medium. These results indicate that the SERS-PCA-based method enables distinction between closely related bacterial strains and thus their identification as different strains. Additionally, the accuracy of such an analysis is very high, making the proposed method consistent and reliable.

#### Comparison of SERS Spectra and PCA Results of *P. gingivalis* and *A. actinomycetemcomitans*

**Figure 3:**
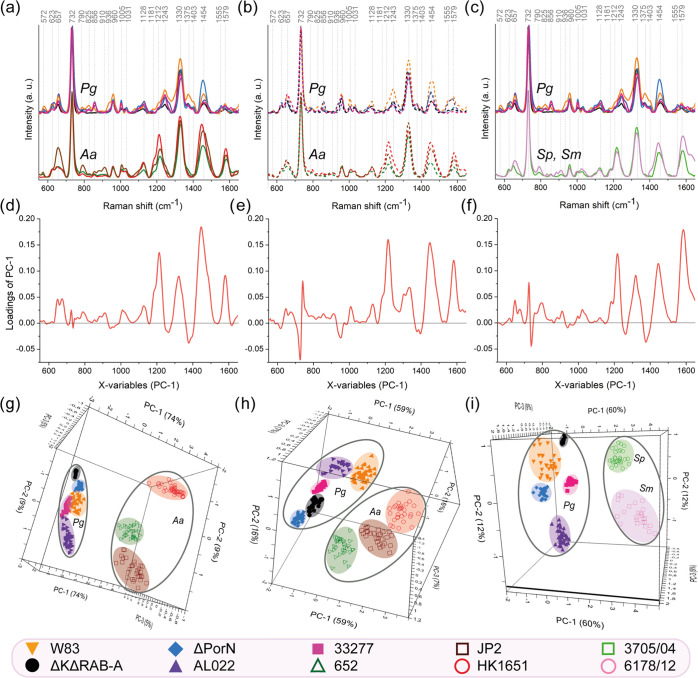
Superimposed SERS spectra (a–c), loading plots (d–f), and PCA (g–i) of *P. gingivalis* (Pg), *A. actinomycetemcomitans* (Aa), and *Streptococcus* spp. (*S. mitis*, Sm, and *S. pseudopneumoniae*, Sp) strains. The loading plots allowed us to determine which bands are responsible for group separation in PCA while analyzing *P. gingivalis* together with *A. actinomycetemcomitans* or *Streptococcus* spp. The images (a, c, d, f, g, i) show results obtained for bacteria grown on agar media, while images (b, e, h) show results obtained for bacteria grown on liquid media. The separation of all three bacterial genera (Pg, Aa, and Sm/Sp) is presented in the Supporting Information (Figure S5).

The proposed technique allows for differentiation between *P. gingivalis* and another common periodontal pathogen, *A. actinomycetemcomitans*, and for discrimination between strains belonging to the same species. This is best visualized by the direct comparison of the plotted-together SERS spectra obtained for the five strains of *P. gingivalis* and the three strains of *A. actinomycetemcomitans* (see Figure 3) both grown on agar plates (Figure 3a) and in liquid cultures (Figure 3b). It can be easily noticed that these two pathogenic species differ in the region between 760 and 940 cm^–1^. While the SERS spectra of all *A. actinomycetemcomitans* strains exhibit weak band at ca. 910 cm^–1^, they are absent in the spectra of *P. gingivalis*. On the contrary, in the SERS spectra of *P. gingivalis*, the bands at ca. 860, 1375, and 1400 cm^–1^ are observed but are absent or very weak in the case of *A. actinomycetemcomitans*. The feature that is also worth noticing is located in the region between 1000 and 1050 cm^–1^. The bands at around 1005 and 1031 cm^–1^ in the spectra of *P. gingivalis* strains are well-separated, while in the spectra of *A. actinomycetemcomitans* they strongly overlap. The last but not least is a strong band located at 1243 cm^–1^, which, is absent in the spectra of the tested *A. actinomycetemcomitans* strains. This lack is compensated by the presence of the strong band at around 1215 cm^–1^ in the SERS spectra of *A. actinomycetemcomitans*, which in the case of *P. gingivalis* can be observed only as a weak shoulder at around 1209 cm^–1^.

All of the abovementioned differences are important for the recognition of well-separated groups in 3D-PCA. The most important SERS signatures responsible for cluster separation are located at around 1212, 1330, 1454, and 1579 cm^–1^, which can be observed in the loading plots (Figure 3d,e). These results apply both for bacteria cultured on agar medium (Figure 3d) and in broth (Figure 3e). The results obtained from 3D-PCA (see Figure 3g,h) indicate that the investigated bacterial strains can be easily divided into two clusters. The first one is grouping together the strains of *P. gingivalis*, while the second one is grouping together the strains of *A. actinomycetemcomitans*. The accuracy of this separation reached 88% in the case of bacteria grown on agar plates and 82% for bacteria from planktonic cultures. This clearly indicates that two tested periodontal pathogens can be easily distinguished in a fast and precise manner.

It is also important to note that all described differences are visible regardless of the type of the used medium, solid or liquid. This, in turn, demonstrates that the color of bacterial colonies formed on agar plates does not influence the obtained spectra. In the case of *A. actinomycetemcomitans*, the cells are pale white both in the liquid and solid media, while *P. gingivalis* wild-type strains exhibit black pigmentation when grown on blood agar and pale white when cultured in a broth. The appearance of the black pigment is due to accumulation of heme on the cell surface.^37,57^ This finding is crucial, as the presence of, e.g., artificial pigmentation of bacterial colonies due to the agar medium supplementation, usually affects the SERS spectra of bacteria.^58^

#### Comparison of SERS Spectra and PCA Results of *P. gingivalis* and Streptococcal Strains

Additionally, we compared the SERS spectra of strains belonging to *P. gingivalis* with those obtained for *S. mitis* 3705/04 and *S. pseudopneumoniae* 6178/12. The first streptococcal species is a common salivary microbiota, while the second one is an emerging respiratory tract pathogen, and thus it can also be detected in human saliva. As mentioned above, the SERS spectra obtained for *P. gingivalis* strains differ from the spectra of streptococcal cells (Figure 3c). These dissimilarities refer especially to the bands at ca. 1185 and 1217 cm^–1^, which are present in the spectra of streptococci and absent in spectroscopic images of *P. gingivalis*. Attention should also be focused on the band at around 1580 cm^–1^, which is very weak for *P. gingivalis* strains and very strong in the case of streptococcal strains. For this reason, the band at 1580 cm^–1^ has the biggest contribution to group separation in PCA, which can be observed in the loading plot of PC-1 versus the Raman shift (see Figure 3f). The other important spectroscopic signatures that distinguish between these two groups of bacteria are located at 1217, 1328, and 1451 cm^–1^. A very similar observation was made when *A. actinomycetemcomitans* was compared to *P. gingivalis* (see Figure 3d,e). Despite this similarity, one should keep in mind that all, even weak, spectroscopic bands are involved in PCA. The results of PCA are shown in Figure 3i. PC-1 together with PC-2 and PC-3 reached 81% of variability, proving once again that SERS coupled with PCA can serve as a fast and reliable differentiation method of bacterial cells.

The PCA and loading plot for all investigated strains belonging to *P. gingivalis* (33277, W83, ΔK/ΔRAB-A, ΔPorN, AL022), *A. actinomycetemcomitans* (HK1651, 652, JP2), and *Streptococcus* spp. (*S. mitis* 3705/04, *S. pseudopneumoniae* 6178/12) are presented in Figure S5.

### Investigation and Chemometric Analysis of Bacterial SERS Spectra Obtained by Applying Fe_2_O_3_@Ag Magnetic NPs and Si/Ag Platforms

#### SERS Spectra and PCA of *P. gingivalis* and *A. actinomycetemcomitans*

**Figure 4:**
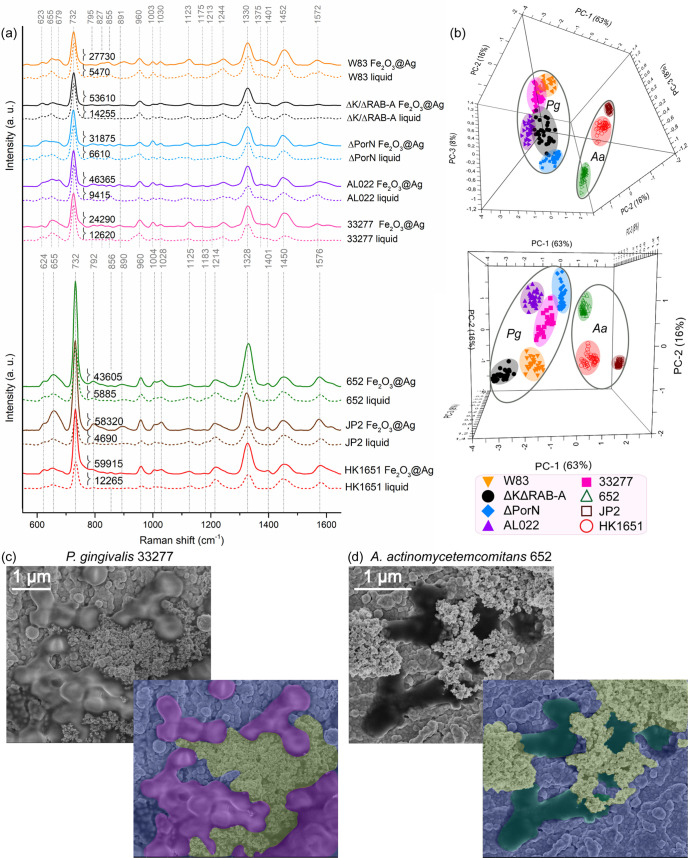
SERS spectra (a), PCA results (b), and SEM images (c, d) obtained for bacterial strains belonging to *P. gingivalis* or *A. actinomycetemcomitans* species. The spectra marked with solid lines show results obtained for bacteria grown in liquid media, which were next rinsed, and dispersed in the saline solution, mixed with Fe_2_O_3_@AgNPs, and magnetically attracted to the Si/Ag SERS substrate in the microfluidic chip. The spectra marked with dashed lines are given for comparison and show results obtained for bacteria grown in liquid media and placed over the Si/Ag SERS substrate (also presented in Figure 2a,2d). The numbers given close to the band at around 730 cm^–1^ represent average counts for this particular band before normalization.

**Scheme 1:**
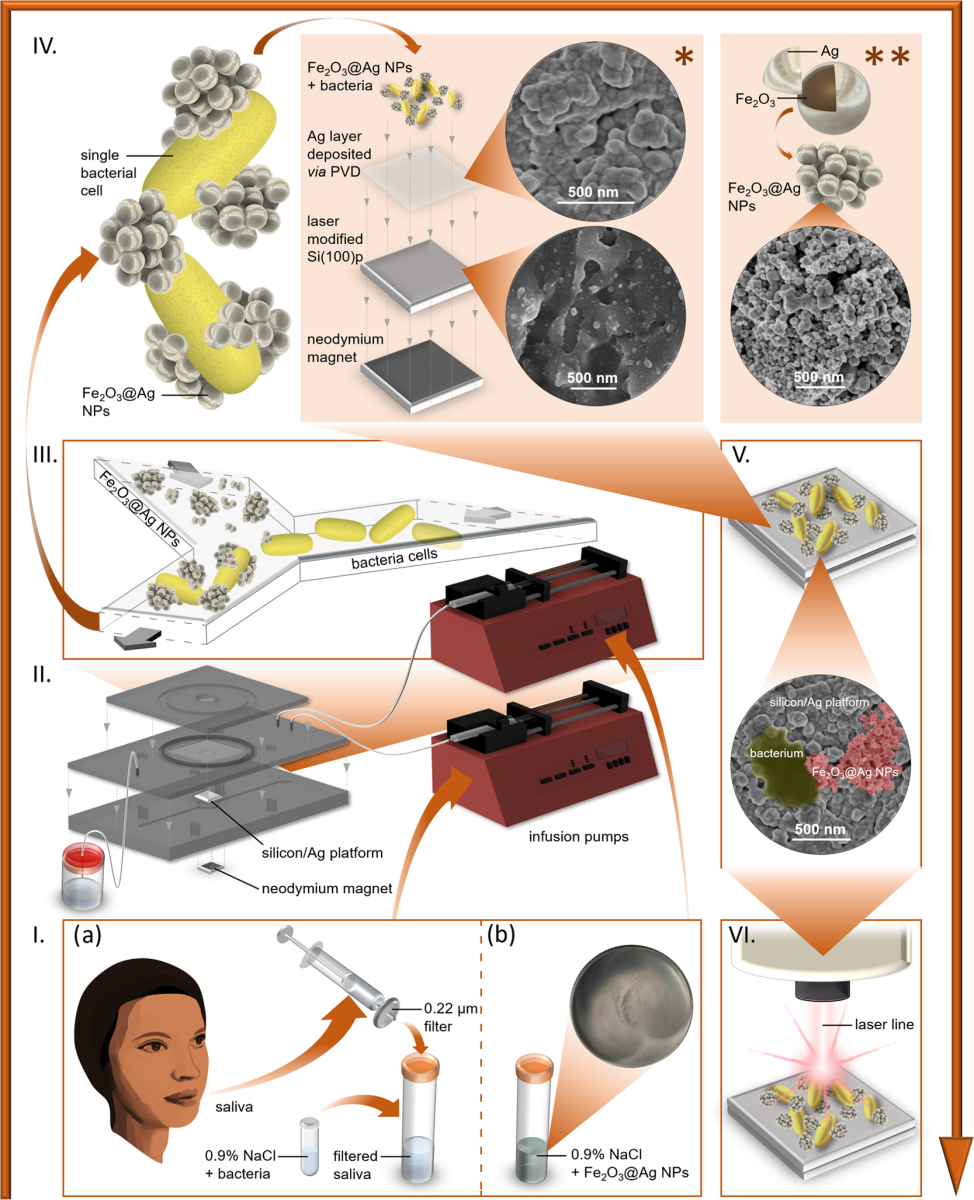
Magnetic-Based Separation of Bacteria from the Clinical Sample The experiment comprised collection, filtration, and inoculation of human saliva (Ia); preparation of the solution of Fe_2_O_3_@AgNPs (Ib); mixing both types of solutions in a microfluidic chip (II); adsorption of bacterial cells to Fe_2_O_3_@AgNPs and formation of bacteria–NP aggregates (III); magnetic attraction of the aggregates to the Si/Ag SERS platform placed over neodymium magnet (* and ** represent the arrangement and SEM images of the Si/Ag substrate and of Fe_2_O_3_@Ag magnetic NPs, respectively) (IV); adsorption of bacteria–NP aggregates to the Si/Ag SERS platform (V); and SERS measurements of the sample (VI).

In Scheme 1, we present the concept of the main experiment. The experiment was performed for two types of samples: bacterial cells mixed with Fe_2_O_3_@AgNPs in (i) saline solution (0.9% NaCl) and (ii) filtered human saliva (for clarity, only the second variant is depicted in the scheme). The first variant was applied to check the functionality of the microfluidic system and whether the presence of an additional and differently prepared SERS substrate affects the spectroscopic image of bacteria. The results of SERS experiments, both for *P. gingivalis* and *A. actinomycetemcomitans*, are presented in Figure 4a. The second variant was performed to verify whether the system can be used to detect bacteria in clinical samples, such as human saliva (see the SERS-Based Bacteria Detection and Identification in Human Saliva section).

As can be noticed in Figure 4a, the similarities between SERS spectra of bacteria obtained by both types of SERS substrates and by applying only the Si/Ag platform are visible with bare eyes (for band assignment, see SERS Spectra of P. gingivalis and SERS Spectra of A. actinomycetemcomitans sections). The biggest difference is the additional Raman signal enhancement, which is visualized by the number of counts for the band at around 730 cm^–1^ for each averaged spectrum (before normalization). This number is up to 1 order of magnitude higher in the case of spectra obtained by applying both Si/Ag and Fe_2_O_3_@Ag SERS substrates than on the bare Si/Ag platform.

Such strong enhancement of the SERS signal is explainable. To elucidate this, one should realize the situation while placing bacterial cells on the SERS platform surface, which is well described in the article by Liu et al.^59^ The authors observed that during standard SERS measurements, cell structures, especially the cell wall and the membrane, do not penetrate the crevices of the SERS substrate, which results in relatively weak enhancement of Raman bands. When the authors used vancomycin, which inhibits peptidoglycan synthesis in the bacterial cell envelope, the cell structures started to penetrate the gaps of the SERS platform. As a consequence, the SERS signal of bacteria was amplified several times. A similar phenomenon, in the physical sense, probably occurs in the case of bacterial cells adsorbed to Fe_2_O_3_@AgNPs and attracted magnetically to the Si/Ag SERS substrate. In such a case, bacteria are affected by strong magnetic fields created by the neodymium magnet, and thus the cell structures easily penetrate into the crevices of the SERS platform.

The sizes of nanostructures building the applied SERS substrates offer another explanation for the observed SERS enhancement. The SEM analysis (Figure S2c,d) revealed that the median diameter of Fe_2_O_3_@AgNPs equals 41 ± 2 nm, while the XRD spectra (Figure S2a) demonstrated that 95.45% of the crystallites of silver present on the Si/Ag platform have an average size of 33.8 nm. Nonetheless, according to the SEM image of the Si/Ag substrate (Figure S2a), these crystallites usually appear as agglomerates of 60–100 nm in diameter and create gaps of similar sizes. Thus, it is postulated that magnetic NPs occupy the free spaces between silver agglomerates on the silicon-based SERS substrate and create additional regions of intense local field enhancement caused by LSPR. Moreover, the described nanostructures are close to the optimal size of NPs for SERS.^60^

Finally, as it can be observed in Figure S6, the Fe_2_O_3_@AgNPs densely surround the cell edges, creating a basket-like structure. This influences the formation of new spots being a source of additional amplification of analytical signals. Last but not least, bacteria, as objects characterized by a large surface area and volume in relation to mentioned spots, show a large cross section for interaction with the recorded backscattered radiation. Therefore, a significant part of the scattered radiation may not reach the detector at all. As NPs locate at the cell edges, the backscattered Raman radiation almost does not interact with the cell structures, in contrast to silver nanostructures of the Si/Ag platform that are situated under the cell.

Due to the strong enhancement of the Raman signal, it is possible to detect relatively low concentrations of bacterial cells in the investigated sample. The obtained limit of detection (LOD) was at the level of 10^3^ cfu/mL (see Figure S7). Such a LOD value is sufficient to detect bacteria in human saliva, where the expected concentration is usually in the range between 10^4^ and 10^8^ cfu/mL.^61,62^

3D-PCA (see Figure 4b) demonstrated the possibility of separating five strains belonging to *P. gingivalis* and three strains of *A. actinomycetemcomitans* (similar to 3D-PCA presented in Figure 3g,h). As can be noticed, PC-1 together with PC-2 and PC-3 is responsible for 87% of the variance in the data when compared with these two species.

#### SEM Images of *P. gingivalis* and *A. actinomycetemcomitans*

The SEM images of *P. gingivalis* 33277 (Figure 4c) and *A. actinomycetemcomitans* 652 (Figure 4d) are presented in duplicates. The top row demonstrates unmodified SEM photographs, while the bottom row shows the same but postproduction colored photographs to highlight individual elements or structures: bacterial cells (marked with pink or green color), Si/Ag platform (marked with blue), and Fe_2_O_3_@AgNPs (marked with yellow). Signal enhancement obtained after simultaneous application of two different SERS substrates indicates that Fe_2_O_3_@AgNPs have a significant contribution to the overall SERS effect.

The SEM images demonstrating bacteria and Fe_2_O_3_@AgNPs on the Si/Ag platform corroborate with our observation presented earlier. As can be noticed, each bacterium is adsorbed to magnetic NPs or connected to other cell(s) (see Figures 4c,d and S6). This is associated with the action of the silver shell of NPs against Gram-negative bacteria, or, more precisely, with the interaction of nano-Ag with sulfur- or phosphorus-containing proteins building the bacterial cell wall.^63^ Moreover, as the core of Fe_2_O_3_@AgNPs exhibits strong magnetic properties, it is possible to attract (via the neodymium magnet) NPs adsorbed to bacterial cells directly from liquid samples to the surface of the Si/Ag SERS substrate.

#### SERS-Based Bacterial Detection and Identification in Human Saliva

**Figure 5:**
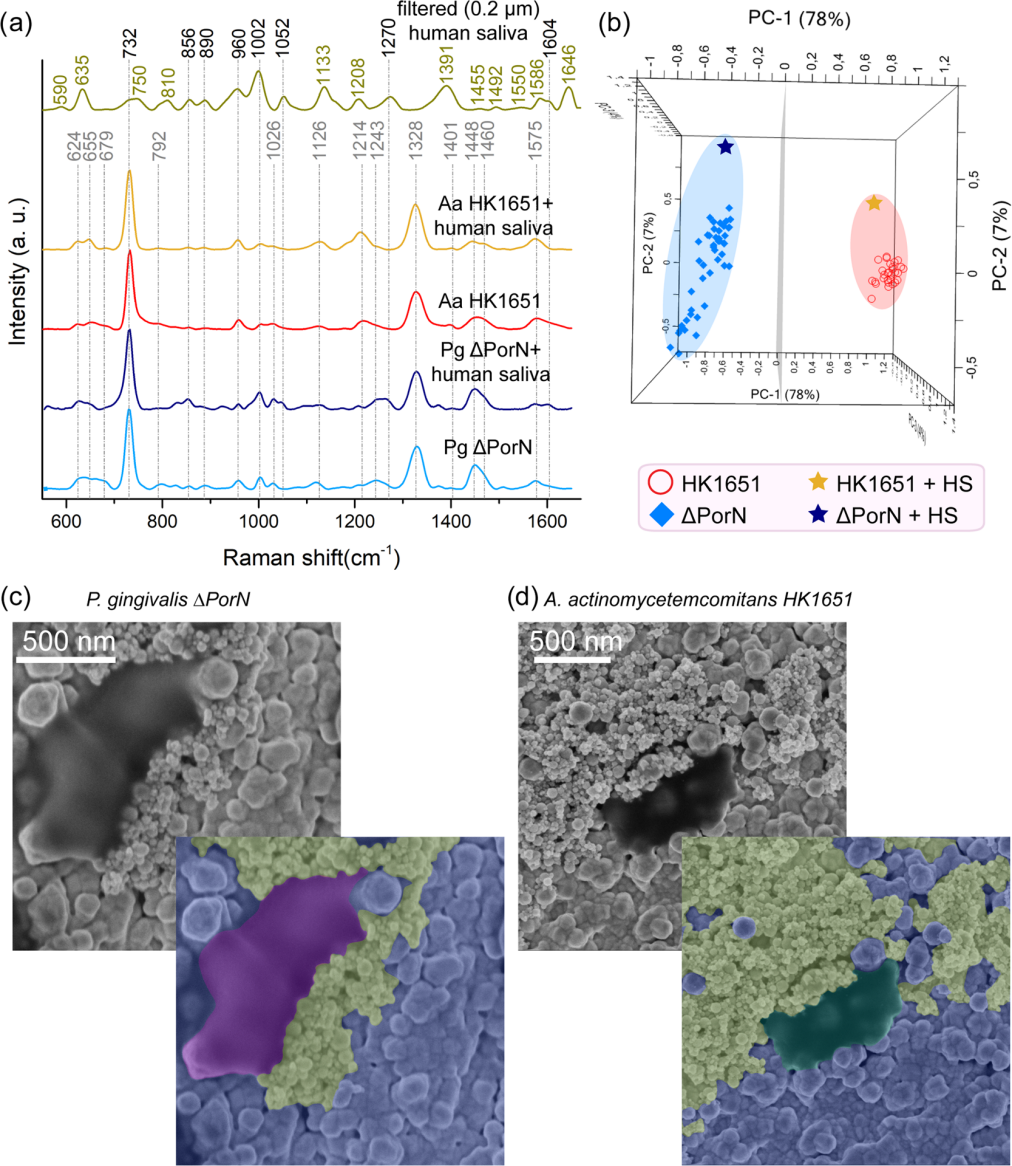
SERS spectra (a), PCA (b), and SEM images (c, d) obtained for two selected bacterial strains belonging to *P. gingivalis* ΔPorN or *A. actinomycetemcomitans* HK165, both mixed with human saliva (HS) and Fe_2_O_3_@AgNPs, and magnetically attracted to the Si/Ag SERS substrate in microfluidic chip. All of the spectra represent bacteria previously grown in liquid media. The spectra of filtered human saliva, *P. gingivalis* ΔPorN, and *A. actinomycetemcomitans* HK1651 in (a) were given for comparison.

The microfluidic system served also to detect single bacterial cells in human saliva. For this purpose, the sample of filtered saliva collected from a healthy volunteer was investigated via the SERS method. Subsequently, two different bacterial strains (*P. gingivalis* ΔPorN and *A. actinomycetemcomitans* HK1651) were separately mixed with 1 mL of filtered human saliva and then with 1 mL of the 0.9% NaCl aqueous solution of magnetic NPs (with a concentration of 1 mg/mL) in a microfluidic chip. The flow in both inlet channels was set at 100 μL/min. The results of this experiment are presented in Figure 5a.

The averaged SERS spectrum of filtered human saliva mixed with Fe_2_O_3_@Ag and placed over the Si/Ag substrate shows various characteristic bands at around 635 (C–C twisting of tyrosine), 750 (ring breathing mode of tryptophan), 810 (uric acid), 856 (ring breathing mode of tyrosine), 890 (structural protein modes, uric acid), 960 (hydroxyapatite, xanthine), 1002 (phenylalanine’s C–C twisting), 1052 and 1133 (C–O stretching in carbohydrates and C–N stretching in proteins), 1208 (tryptophan, tyrosine, phenylalanine–protein assignment), 1270 (amide III band in proteins), 1391 (CH rocking), 1586 (phenylalanine, hydroxyproline, hypoxanthine), 1604 (C=C in-plane bending mode of phenylalanine and tyrosine), and 1646 cm^–1^ (amide I).^48,64,65^ These results highly correspond with the results presented in the literature^65^ (slight differences may be attributed to the filtration process, which was applied in the present study).

Despite the fact that the spectrum of human saliva has many bands, their contribution in the spectra of bacteria mixed with saliva is almost unnoticeable or minute, which can be observed in Figure 5a. The differences are limited to a slight increase in the intensity of the bands at around 1126 and 1214 cm^–1^ for *A. actinomycetemcomitans* HK1651 mixed with saliva compared to *A. actinomycetemcomitans* HK1651 suspended in the saline solution, which is probably because of the fact that the spectrum of filtered human saliva exhibits bands almost in the same regions (1133 and 1208 cm^–1^). In the case of *P. gingivalis* ΔPorN mixed with saliva, one can notice the increase in the intensity of the bands at around 856 and 1002 cm^–1^ in comparison with *P. gingivalis* ΔPorN suspended in saline. Moreover, additional weak bands at ca. 1052, 1270, and 1604 cm^–1^ appeared. All mentioned bands can be assigned to compounds present in human saliva.

To demonstrate that the SERS-based technique can serve as a method for bacterial identification in clinical samples, the spectra of human saliva samples containing *P. gingivalis* ΔPorN (ΔPorN + HS) or *A. actinomycetemcomitans* HK1651 (HK1651 + HS) were averaged and analyzed via 3D-PCA together with measurements performed for *P. gingivalis* ΔPorN and *A. actinomycetemcomitans* HK1651 in saline. The results shown in Figure 5b demonstrate that yellow (ΔPorN + HS) and navy blue (HK1651 + HS) stars, representing the mentioned averaged spectra, are located in close proximity to the groups representing the SERS measurement of *P. gingivalis* ΔPorN and *A. actinomycetemcomitans* HK1651 in saline solution, respectively. The PC-1 reached 78% of total variance and together with PC-2 and PC-3 the value of 89% was obtained. This clearly proves that different bacterial species can be easily detected in clinical samples such as saliva, distinguished from each other, and taxonomically identified by the SERS-PCA-based technique.

The SEM images of *P. gingivalis* ΔPorN (Figure 5c) and *A. actinomycetemcomitans* HK1651 (Figure 5d) reveal singular bacterial cells adsorbed to magnetic NPs and the Si/Ag platform. The cells were previously suspended in human saliva, mixed with Fe_2_O_3_@AgNPs in the microfluidic chip, and then attracted to the Si/Ag SERS substrate via the neodymium magnet.

## Conclusions

Here, we described the development of an easy, fast, and sensitive method for identification of pathogens causing periodontal disease. We presented that it is possible to obtain the intense and unique SERS spectra of bacterial cells in ca. 45 s, using only Si/Ag substrates and a portable Raman spectrometer. Moreover, PCA allowed us to separate the SERS spectra of bacteria belonging to different species (82, 88, and 91% of accuracy, depending on the analyzed sample). Such good separation coupled with very fast identification is an undisputed advantage of the SERS technique. The similar effect was also obtained for bacterial strains belonging to the same species.

Additionally, we proved that it is possible to detect and identify *P. gingivalis* and *A. actinomycetemcomitans* strains in clinical samples such as human saliva. For this purpose, the Si/Ag platform, Fe_2_O_3_@Ag magnetic NPs, and the microfluidic chip were applied. As a result, the formation of bacteria–NP aggregates, magnetic separation, and Raman signal enhancement of bacteria were achieved. The obtained SERS signal was relatively strong, which is very beneficial, especially when detecting low concentrations of bacteria in clinical samples. Additionally, the PCA performed for bacteria suspended in human saliva allowed us to separate *P. gingivalis* from *A. actinomycetemcomitans* strains with 89% accuracy. Furthermore, the sample can be prepared in a rapid and label-free manner. There is also no need for expensive reagents. This, in turn, may lead to lowering the costs for the healthcare industry by early detection of ongoing infection and stopping illness development. The identification of different strains is additionally beneficial as strains of the same species often have different virulence potentials (described as pathotypes or serotypes) and therefore may pose various threats to human health.

Rapid detection and identification of *P. gingivalis* offered by the SERS-PCA technique are potentially of great importance for human health, as they provide unmatched diagnostic power essential for prevention of the development of chronic infection of periodontal tissues, which could lead to tooth loss, and comorbidities such as cardiovascular diseases, rheumatoid arthritis, and some cancer pathologies. Recent reports suggest that *P. gingivalis* may also be involved in Alzheimer’s disease. Thus, in the near future, we plan to apply the SERS-PCA-based technique for *P. gingivalis* detection in the blood and the cerebrospinal fluid. This enables early and appropriate patient treatment to reduce the incidence of diseases associated with *P. gingivalis* proliferation in the periodontal pockets and thus its systemic dissemination.

## Supporting Information Available

Generation of *P. gingivalis* AL022 (ΔPGN_1642) mutant strain and its genetic analysis; analysis of the diameter of SERS-active nanostructures based on SEM images or XRD spectra; detailed scheme of the microfluidic chip used for magnetic separation of bacterial cells from liquid samples; superimposed SERS spectra of all investigated *P. gingivalis* and *A. actinomycetemcomitans* strains cultured both on agar and in liquid media; the PCA and loading plots of *P. gingivalis* together with *A. actinomycetemcomitans* and *Streptococcus* spp.; additional SEM images presenting *P. gingivalis* and *A. actinomycetemcomitans* adsorbed to Fe_2_O_3_@AgNPs and attracted to the Si/Ag platform via the neodymium magnet; and limit of detection (LOD) measured for bacterial samples after applying the SERS-microfluidic-based method (PDF)
