# Supplementary material for: In Search of Spectroscopic Signatures of Periodontitis: A SERS-Based Magnetomicrofluidic Sensor for Detection of Porphyromonas gingivalis and Aggregatibacter actinomycetemcomitans
Source: ACS Sens. 2021 Apr 1;6(4):1621–35. doi: 10.1021/acssensors.1c00166 (PMC8155661; doi:10.1021/acssensors.1c00166)
Supplement: Supplementary file 1 — se1c00166_si_001.pdf [file se1c00166_si_001.pdf]

Supporting Information:

In search of spectroscopic signatures of periodontitis: the SERS-based magneto-microfluidic sensor for detection of *Porphyromonas gingivalis* and *Aggregatibacter actinomycetemcomitans*

Evelin Witkowska[a]*, Anna Maria Łasica[b†], Krzysztof Niciński[a†], Jan Potempa[c,d] and Agnieszka Kamińska[a]*

[a] Institute of Physical Chemistry, Polish Academy of Sciences, Kasprzaka 44/52, 01-224 Warsaw, Poland

[b] University of Warsaw, Faculty of Biology, Institute of Microbiology, Department of Bacterial Genetics, Miecznikowa 1, 02-096 Warsaw, Poland

[c] Department of Microbiology, Faculty of Biochemistry, Biophysics, and Biotechnology, Jagiellonian University, Gronostajowa 7, 30-387 Krakow, Poland

[d] Oral Immunology and Infectious Diseases, University of Louisville School of Dentistry, 501 S. Preston St, Louisville, Kentucky, USA

e-mail: ewitkowska@ichf.edu.pl; akamin@ichf.edu.pl

* Corresponding authors

† These authors contributed equally to this work

<u>The generation of *Porphyromonas gingivalis* AL022 (*ΔPGN_1642*) mutant strain.</u>

- **Bacterial strains, culture media and growth conditions**

Porphyromonas gingivalis ATCC 33277 (wt, parental strain) was grown in enriched BD BBL trypticase soy broth (eTSB per liter: 30 g trypticase soy broth, 5 g yeast extract, 5 mg hemin, pH 7.5; 0.5 g L-cysteine and 2 mg menadione) or on eTSB blood agar (eTSB medium containing 1.5 % agar and 5 % defibrinated sheep blood) at 37°C in an anaerobic condition provided with Anoxomat Mark II (90 % nitrogen, 5 % carbon dioxide and 5 % hydrogen). *P. gingivalis* AL022 (*ΔPGN_1642*) mutant growth media were additionally supplemented with erythromycin at 5 µg/ml. *Escherichia coli* strain TG1, used for mutagenesis suicide plasmid

construction, was grown aerobically at 37°C in Luria–Bertani (LB) medium and on 1.5 % agar LB plates supplemented with ampicillin at 100 µg/ml when needed.

- **DNA manipulation and visualization**

All basic DNA manipulation and visualization techniques as well as *E. coli* transformation were previously described by Sambrook et al.[1] or were conducted according to the manufacturer's instructions. Polymerase chain reactions (PCR) were carried up with Phusion™ High-Fidelity DNA Polymerase (Thermo Scientific™) under standard conditions. Oligonucleotide primer synthesis (sequences given in Table S1) and DNA sequencing were performed by Genomed, Warsaw, Poland. Restriction enzymes (Fast Digest) and T4 ligase were purchased from Thermo Scientific™. DNA was visualized in 0.8-1% agarose gels (Prona) with a use of Midori Green Advance/Direct loading dye (NIPPON Genetics).

- **Mutagenesis suicide plasmid construction**

Mutagenesis plasmid was constructed so that it could be used for double-crossover homologous recombination. Therefore, two - 0.7 kb and 0.9 kb flanking regions on either side of the *PGN1642* (*PGN_RS07810*) were amplified from genomic DNA of *P. gingivalis* 33277 by PCR with primer pairs PGN_1642_up_EcoRI / PGN_1642_up_BamHI and PGN_1642_dw_SphI / PGN_1642_dw_SalI, respectively (Table S1). Amplified fragments were subsequently cloned into pAL35 vector – a pUC19 derivative with *P. gingivalis* erythromycin-resistance cassette – *ermF*,[2] flanked with SalI/BamHI restriction enzymes. Resulting mutagenesis plasmid designated pAL53 was sequenced to confirm its correct construction.

- ***P. gingivalis* gene mutagenesis**

P. gingivalis deletion mutant was generated with a use of allele exchange method as previously described for other genes;[3] pAL53 suicide plasmid was introduced by electroporation into electrocompetent wild-type *P. gingivalis* cells prepared according to protocol included in.[4] Obtained clones were selected on erythromycin plates and double-crossover genomic recombination was confirmed by PCR specific for mutated region (Figure S1a, b, Table S1) and DNA sequencing of pertinent manipulated region.

- **Exclusion of mutation polar effect**

Analysis of *P. gingivalis* 33277 genome fragment encoding *PGN_1642* revealed that the gene of interest may be a part of larger transcriptional unit *PGN_1643-1642-1641-1640*. Therefore, we checked if replacement of *PGN_1642* gene with erythromycin cassette (*ermF*) didn't alter transcription (polar effect) of two following genes *PGN_1641* and *PGN_1640*. Total mRNA was isolated with TRIzol Reagent (Thermo Scientific) from *P. gingivalis* AL022 mutant and reverse-transcribed with SuperScript III First-Strand Synthesis System (Invitrogen) using universal primer provided. Resulting cDNA was then used as a template for PCR reactions detecting the presence of transcripts for *PGN_1641* and *PGN_1640* genes (Figure S1a, c). Experiment confirmed no polar effect of introduced mutation in AL022 strain. Transcripts for both genes (*PGN_1641* and *PGN_1640*) were present in AL022 strain (RT+) and absent in reactions performed without reverse transcriptase (RT-; negative control) shown on Figure S1c.

Genomic DNA (g) of AL022 strain was used as positive control template. Primers utilized for this experiment are listed in Table S1 and marked on a scheme (Figure S1a).

Table S1. Oligonucleotides used in this study

No	Name	Sequence	Restriction site*
	**Oligonucleotides used for mutagenesis**		
1	**PGN_1642_up_EcoRI**	TATGAATTCTGGAACAATTCTCGAGCG	EcoRI
2	**PGN_1642_up_BamHI**	GCCGGATCCATGTACTGACGATCATAC	BamHI
3	**PGN_1642_dw_SphI**	ATTGCATGCGATGCCATCGGCCATCTC	SphI
4	**PGN_1642_dw_SalI**	TATGTCGACTGCGCATCGCTGTGGATC	SalI
5	**PGN_1644_Fwd**	CTCGCGCCACGTTTTTAGG	None
6	**PGN_1641_Rev[#]**	CTGAATAGGTGAGGACTCG	None
7	**ErmFR**	TCTATGATGTTGCAAATACCG	None
8	**ErmF_seq_fwd2**	TTCTGGGAGGTTCCATTG	None
	**Oligonucleotides used for checking mutation polar effect**		
9	**PGN_1640_Nde**	CCAACCATATGCGTAAAC	NdeI
10	**PGN_1640_Xho**	GGTCATATTCAGAAAGATCC	XhoII
11	**PGN_1642_dw_seq4**	ATCAAGCAGGGACGGAAG	None

* restriction site sequence was underlined; not all sites used for cloning purposes

[#] also used for checking mutation polar effect

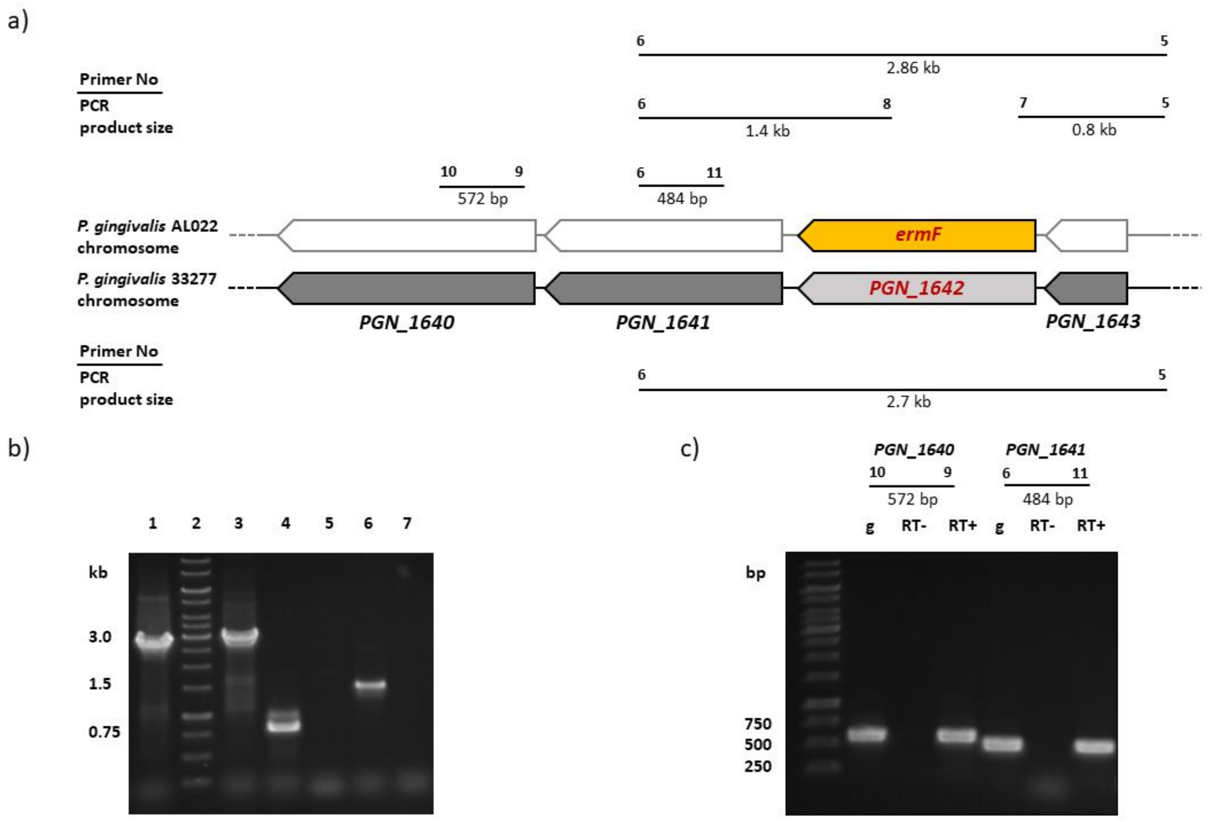

Figure S1. Genetic analysis of *P. gingivalis* AL022 (*ΔPGN_1642*) mutant strain.

a) Schematic view of *P. gingivalis* genome region containing *PGN_1642* and primers used for confirmation of mutagenesis and analysis of polar effect.

b) Confirmation of correct genetic construction of AL022 strain. Lane 1 – PCR reaction on chromosomal DNA of the wild-type *P. gingivalis*, primers no 5 and 6; lane 2 – DNA marker; lanes – 3-7 - PCR reactions on chromosomal DNA of *P. gingivalis* AL022. Lane 3 – primers no 5 and 6; Lanes 4 - 7 – reactions with *ermF* gene primers confirming the presence of antibiotic cassette in a place of *PGN_1642* gene and their alike forward orientation; lane 4 – primers no 5 and 7; lane 5 – primers no 6 and 7 (negative), lane 6 – primers no 6 and 8, lane 7 – primers no 5 and 8 (negative).

c) Analysis of possible polar effect of *PGN_1642* gene replacement in AL022 strain. (g) – genomic DNA of AL022 used as a template (positive control); (RT-) - PCR reaction on cDNA prepared without reverse transcriptase (negative control); (RT+) - PCR reaction on cDNA prepared with reverse transcriptase.

The sizes of SERS-active nanostructures

a)

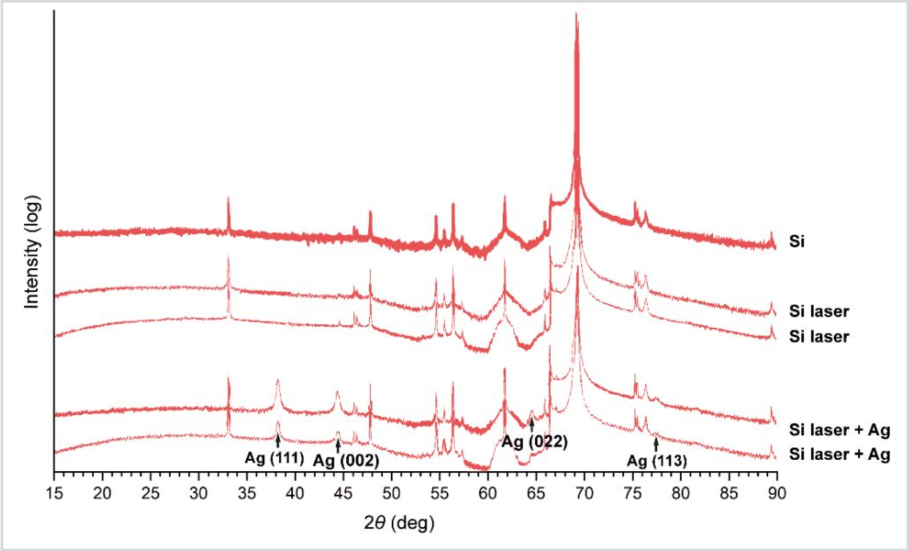

b)

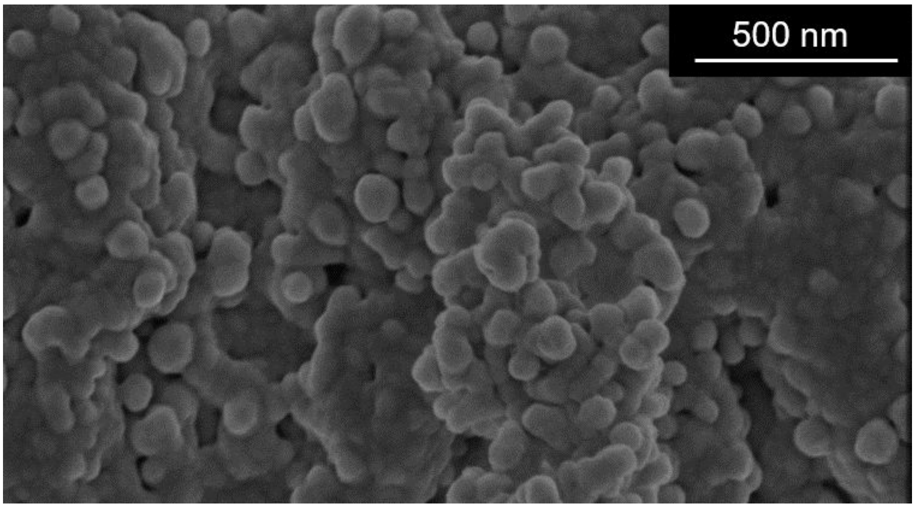

c)

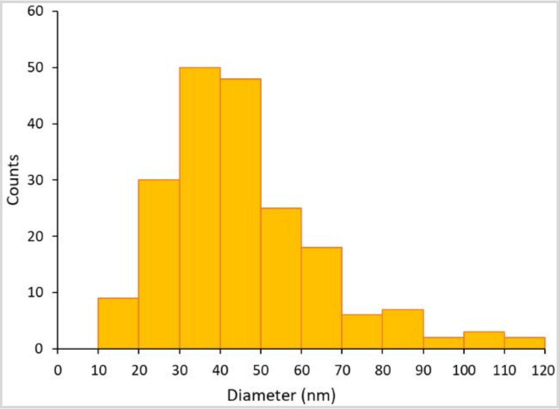

d)

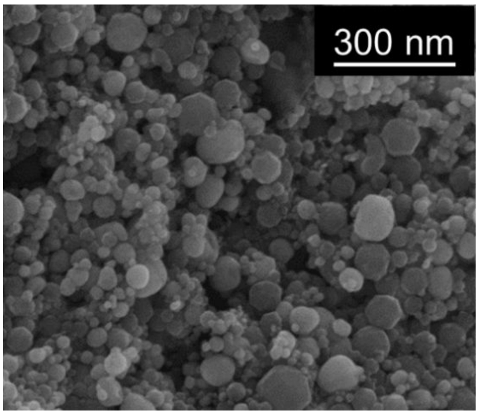

Figure S2. The analysis of the diameter of SERS-active nanostructures based on SEM images or XRD spectra; the X-ray diffraction (XRD) of pure silicon (Si), silicon after laser ablation (Si laser) and silicon after laser ablation and deposition of 100 nm layer of silver (Si laser + Ag) (a); the SEM image of Si/Ag platform (b); the histogram of the diameters of Fe_2O_3@Ag NPs based on 200 measurements in ImageJ (c); the SEM image of Fe_2O_3@Ag NPs (d).

The construction of microfluidic chip

a)

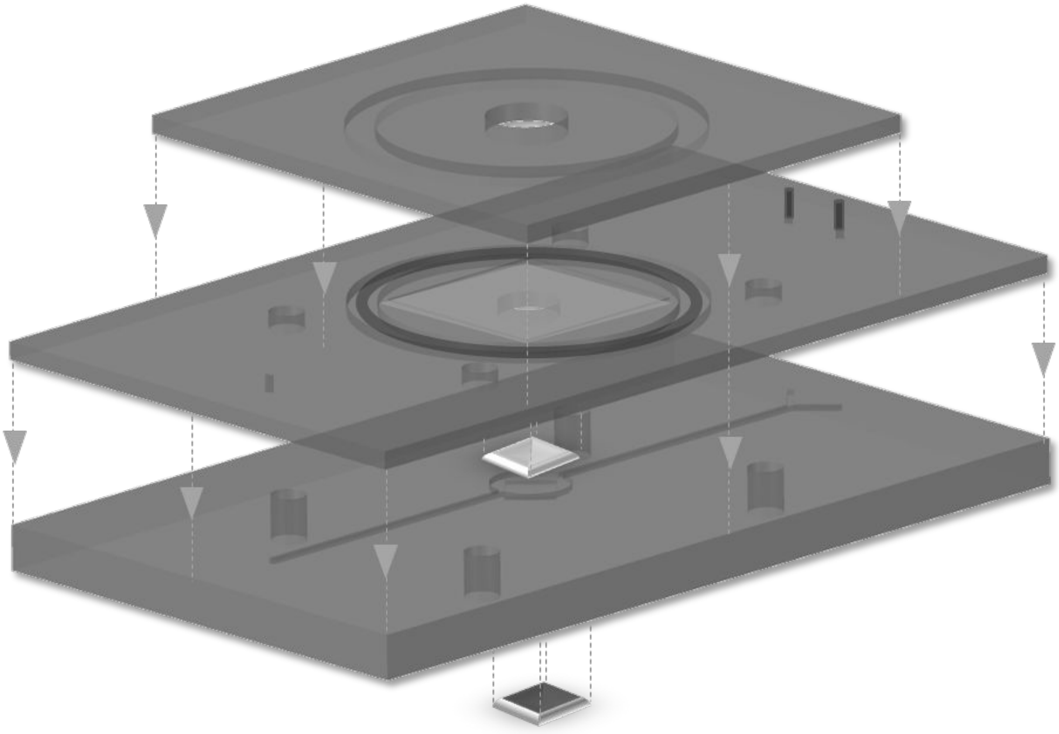

b)

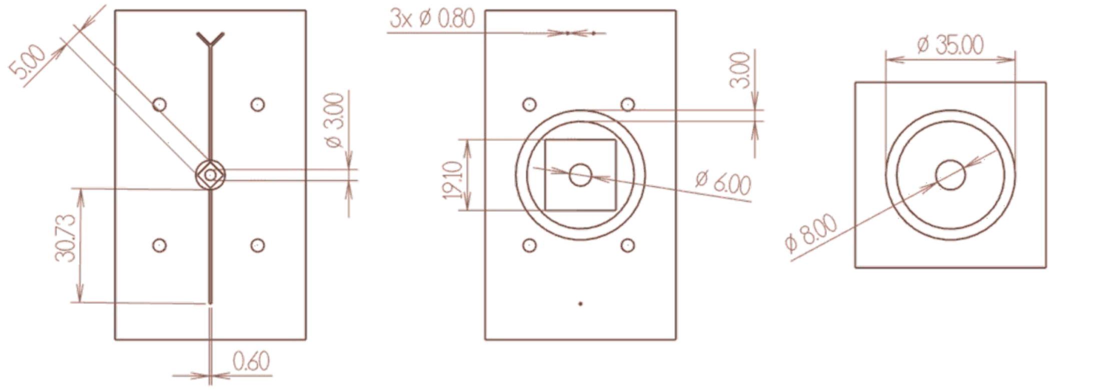

Figure S3. Microfluidic chip used for magnetic separation of bacterial cells from liquid samples; the scheme presenting the view from the angle (a) and technical drawing presenting the view from the top, with given dimentions (b).

<u>The superimposed and avaraged SERS spectra of *Porphyromonas gingivalis* and *Aggregatibacter actinomycetemcomitans*</u>

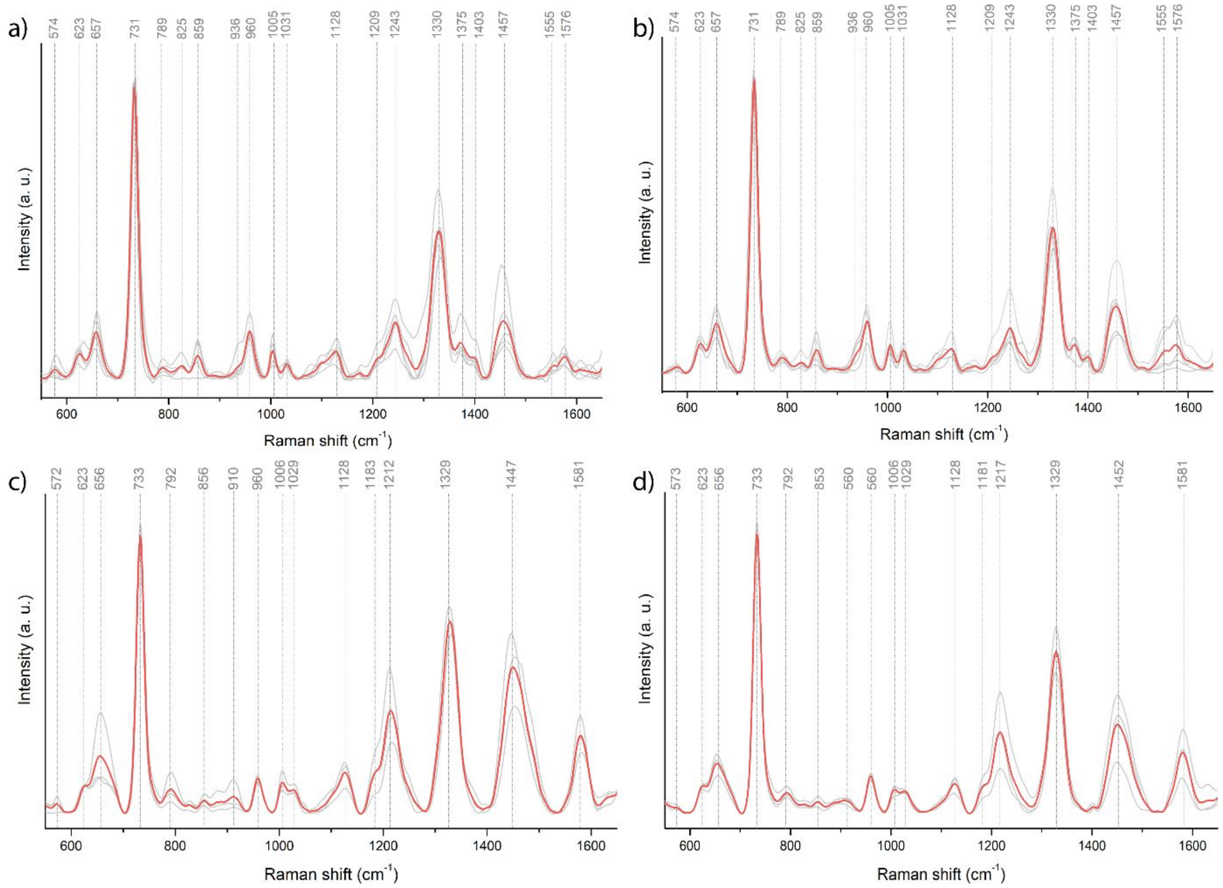

Figure S4. The superimposed SERS spectra (grey) of all investigated *P. gingivalis* (a, b) and *A. actinomycetemcomitans* (c, d) strains, measured on Si/Ag SERS platform. The orange spectra demonstrate the average results for strains plotted together. Images (a, c) show results obtained for bacteria grown on agar media, while images (b, d) – on liquid media.

<u>The PCA and loadings plot of *P. gingivalis* together with *A. actinomycetemcomitans* and *Streptococcus* spp.</u>

a) b)

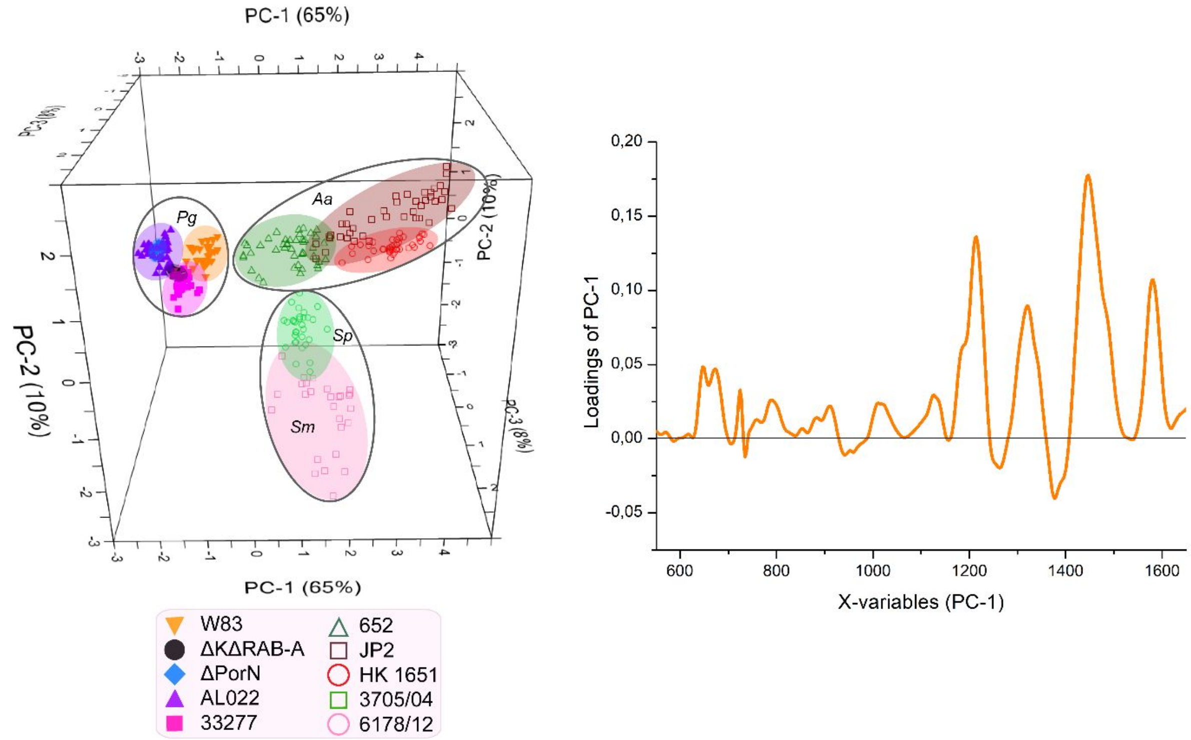

Figure S5. The PCA (a) and loadings plot (b) for ten strains belonging to *P. gingivalis* (33277, W83, ΔK/ΔRAB-A, ΔPorN, AL022), *A. actinomycetemcomitans* (HK1651, 652, JP2) and *Streptococcus* spp. (*S. mitis* 3705/04, *S. pseudopneumoniae* 6178/12).

The SEM images of *Porphyromonas gingivalis* and *Aggregatibacter actinomycetemcomitans* adsorbed to Fe_2O_3@Ag NPs and Si/Ag substrate

a)

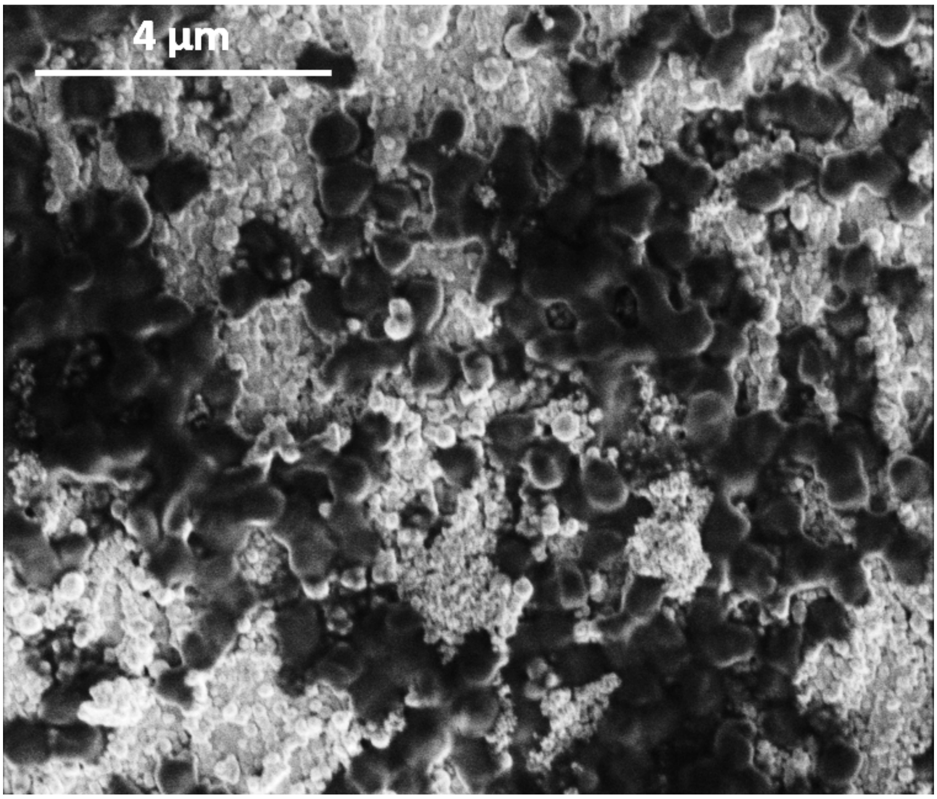

b)

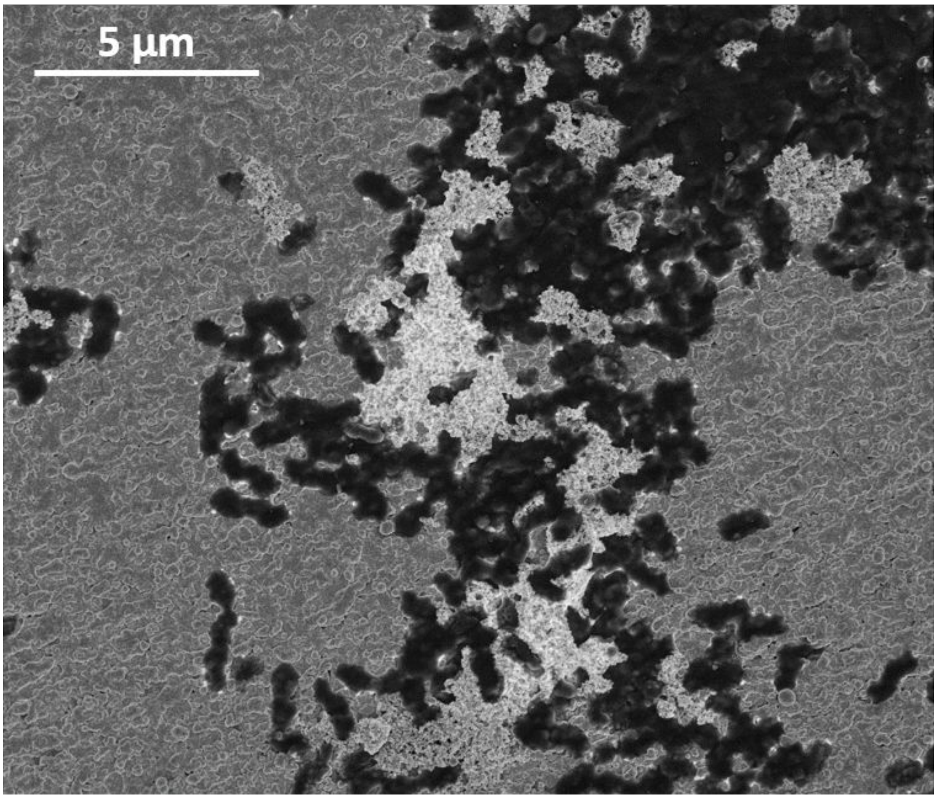

Figure S6. The SEM images of *P. gingivalis* (a) and *A. actinomycetemcomitans* (b) adsorbed to Fe_2O_3@Ag NPs and attracted to Si/Ag platform *via* neodymium magnet.

__**Limit of detection (LOD)**__

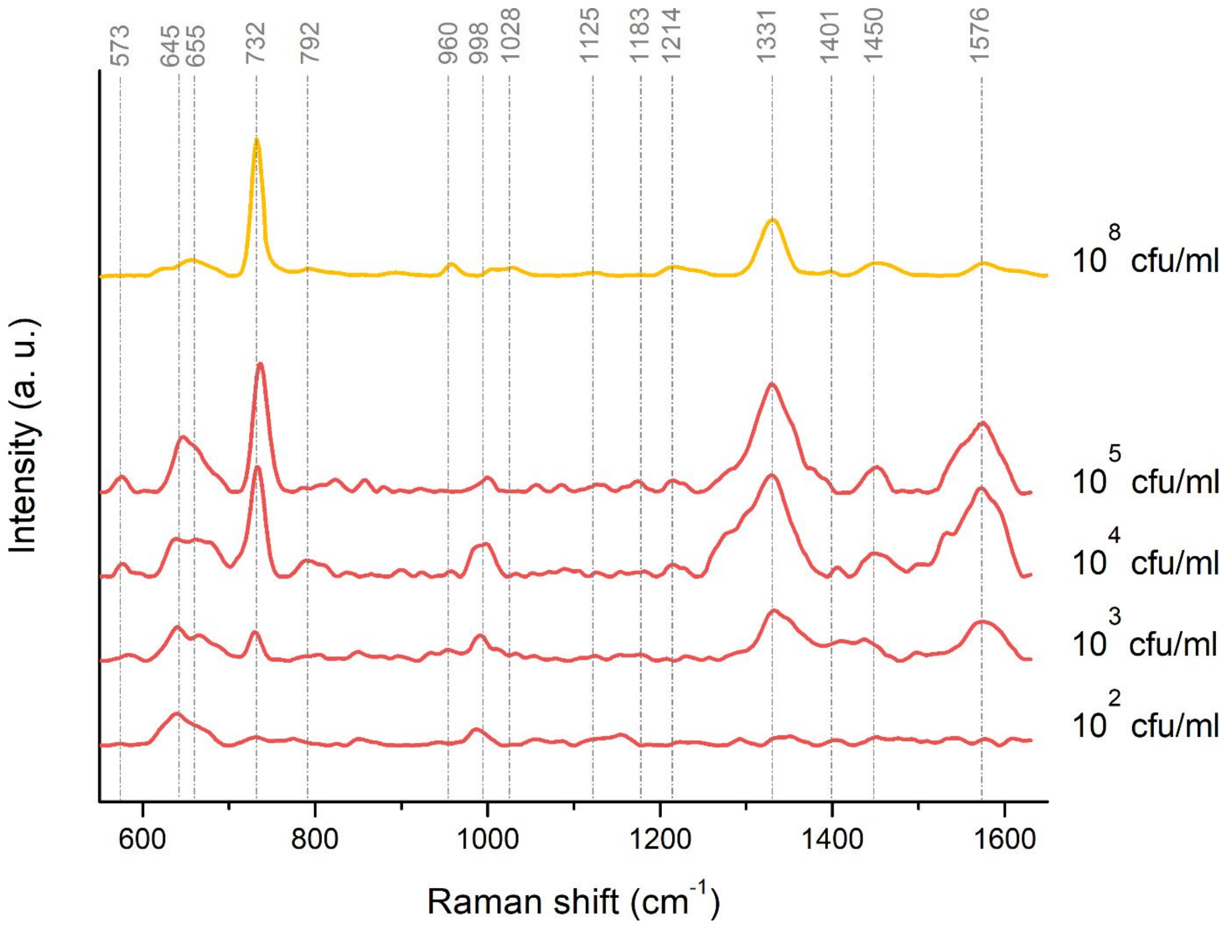

Figure S7. The limit of detection (LOD) of bacterial cells for the proposed SERS-microfluidic-based method. In order to verify the LOD, 1 ml of each of the following concentrations of *A. actinomycetemcomitans* 652: 10^5, 10^4, 10^3, and 10^2 cfu/ml (red spectra) was placed in the microfluidic device. The samples were prepared by serial dilution of the initial sample which was adjusted for 0.5 McFarland turbidity standards (10^8 colony forming units/ml).[5] The spectrum of *A. actinomycetemcomitans* 652 (dark yellow), collected during standard measurement and described in the main manuscript, was given for comparison.

References:

1. Sambrook, J.; Russell, D. W., *Molecular cloning : a laboratory manual. 3*. Cold Spring Harbor Laboratory Press: **2001**.

2. Fletcher, H. M.; Schenkein, H. A.; Morgan, R. M.; Bailey, K. A.; Berry, C. R.; Macrina, F. L., Virulence of a *Porphyromonas gingivalis* W83 mutant defective in the prtH gene. *Infect. Immun.*, **1995,** *63* (4), 1521-1528.

3. Nguyen, K. A.; Travis, J.; Potempa, J., Does the importance of the C-terminal residues in the maturation of RgpB from *Porphyromonas gingivalis* reveal a novel mechanism for protein export in a subgroup of Gram-Negative bacteria? *J. Bacteriol.* **2007,** *189* (3), 833-843.

4. Belanger, M.; Rodrigues, P.; Progulske-Fox, A., Genetic manipulation of *Porphyromonas gingivalis*. *Curr. Protoc. Microbiol.*, **2007**, *Chapter 13*, Unit13C.2.

5. Emani. S.; Gunjiganur, G.V.; Mehta, D.S. Determination of the antibacterial activity of simvastatin against periodontal pathogens, *Porphyromonas gingivalis* and *Aggregatibacter actinomycetemcomitans*: An in vitro study. *Contemp. Clin. Dent.* **2014**, *5* (3), 377-382.
